# Elevated risk of acute epiglottitis in patients with chronic obstructive pulmonary disease: A nationwide cohort study

**DOI:** 10.1371/journal.pone.0273437

**Published:** 2022-08-19

**Authors:** Shu-Yi Huang, Cheng-Ming Hsu, Yao-Hsu Yang, Yuan-Hsiung Tsai, Ming-Shao Tsai, Geng-He Chang, Chia-Yen Liu, Yi-Chan Lee, Ethan I. Huang, Yao-Te Tsai

**Affiliations:** 1 Department of Pulmonary and Critical Care Medicine, Chang Gung Memorial Hospital, Chiayi, Taiwan; 2 Department of Otorhinolaryngology-Head and Neck Surgery, Chang Gung Memorial Hospital, Chiayi, Taiwan; 3 Health Information and Epidemiology Laboratory, Chang Gung Memorial Hospital, Chiayi, Taiwan; 4 Department of Traditional Chinese Medicine, Chang Gung Memorial Hospital, Chiayi, Taiwan; 5 Department of Radiology, Chang Gung Memorial Hospital, Chiayi, Taiwan; 6 Department of Otorhinolaryngology-Head and Neck Surgery, Chang Gung Memorial Hospital, Keelung, Taiwan; University of Colorado School of Medicine, UNITED STATES

## Abstract

**Objective:**

In individuals with epiglottitis, chronic obstructive pulmonary disease (COPD) is a common comorbidity; however, the impact of COPD under such circumstances is not well documented. Therefore, we performed this population-based study to determine whether, in adults, COPD is a risk factor for epiglottitis.

**Methods:**

In this retrospective matched-cohort study, data obtained from the Taiwan National Health Insurance Research Database were analyzed. We identified all patients newly diagnosed as having COPD in 2000–2011 and performed frequency matching and propensity-score matching for every patient with COPD individually to another patient without a COPD diagnosis. We used epiglottitis occurrence as the study endpoint, and we investigated the hazard ratio of epiglottitis by using the Cox proportional hazards model after adjustment for potential confounders.

**Results:**

In the frequency matching, the cumulative epiglottitis incidence was significantly higher (*p* = 0.005) in the COPD cohort. According to the adjusted Cox proportional hazard model, COPD exhibited a significant association with elevated epiglottitis incidence (adjusted hazard ratio: 1.76; 95% confidence interval: 1.15–2.70, *p* = 0.009). Similar trend was observed in the propensity-score matching analysis (adjusted hazard ratio: 1.50; 95% confidence interval: 0.99–2.29, *p* = 0.057). Our subgroup analysis revealed COPD to be an epiglottitis risk factor in male patients and those aged 40–64 years.

**Conclusions:**

This is the first nationwide matched-cohort research to examine the association of COPD with epiglottitis. Our results revealed that COPD may be a potential risk factor for epiglottitis; thus, clinicians should be mindful of the potential increased risk of epiglottitis following COPD.

## Introduction

Epiglottitis refers to rapid-onset inflammation over the supraglottic area, including the epiglottis, aryepiglottic folds, and arytenoids. Patients with epiglottitis typically require aggressive treatment and intensive care because of the potential acute and life-threatening airway compromise [1,2] as well as severe complications, including sepsis, mediastinitis [3], and necrotizing fasciitis [4]. When epiglottitis is suspected, lateral neck plain film radiography, ultrasonography, and flexible pharyngo-laryngoscopy are useful tools for early diagnosis and timely treatment [5]. Most epiglottitis is caused by bacterial infection, and other reported causes include viral and fungal infections as well as non-infectious etiologies [6]. Nevertheless, in spite of detailed examinations such as the throat or blood cultures, a specific pathogen can be detected in only 10%–25% of patients with epiglottitis [7]. Since inclusion of *Haemophilus influenzae* in a vaccination program for children, the incidence of epiglottitis has decreased in this demographic [8]. Modern epiglottitis has thus seemingly become an adult disease [9], and more than half of adults with epiglottis have underlying comorbidities such as cardiovascular disease, autoimmune disease, and diabetes mellitus [7,10,11].

Globally, the prevalence of chronic obstructive pulmonary disease (COPD) is approximately 10%, and the disease is the fourth most common cause of death [12]; it is characterized by progressive airway inflammation and non–fully reversible airflow limitation [13]. An increasing body of evidence suggests that COPD is not only a chronic small airway inflammatory disease but also a complex systemic disease [14], and patients with COPD frequently exhibit impaired immunity to respiratory pathogens and chronic systemic inflammation that contribute to their immunosuppressive status and susceptibility to diverse infections [15–18]. Studies have indicated that patients with epiglottitis frequently have underlying respiratory tract disease, such as COPD and asthma, which considerably exacerbate the clinical course of epiglottitis possibly because of the preceding impaired pulmonary function and immune dysregulation [9,19,20]. In a retrospective cohort study that included 6,072 patients with severe epiglottitis requiring intensive care, Suzuki et al. observed that 15.4% of the patients had prior chronic pulmonary disease [20]. The pathophysiological elements involved in the dysregulated inflammation and immune response may contribute to the association between epiglottitis and COPD [17,21]. However, whether patients with COPD have increased risk of epiglottitis has not been investigated because of the rarity of the disease as well as the dearth of comparison groups for examining the statistical significance of findings. The absence of related evidence hampers the formulation of useful clinical guidance. The hypothesis of this study is that an association exists between COPD and increased epiglottitis risk in adults. We examined this hypothesis by first recruiting patients with COPD from the National Health Insurance Research Database (NHIRD) and then comparing the risk of epiglottitis with that in cohorts who did not have COPD.

## Material and methods

### Ethics statement

This retrospective research was conducted in compliance with the Declaration of Helsinki, and our study protocol was ratified by Chang Gung Memorial Hospital’s Institutional Review Board (IRB; No. 201901122B0C502). Patient data in the NHIRD are deidentified to respect and ensure patient privacy; therefore, the informed consent requirement was waived. The confidentiality of enrolled patients’ personal data is guaranteed by the Chang Gung Medical Foundation IRB and the National Health Insurance (NHI) administration.

### Study design and data source

We conducted the current nationwide matched-cohort study by employing data from Taiwan’s NHIRD. The NHI program, a compulsory and single-payer insurance plan, was established by the Taiwanese government in March 1995. As of 2018, the program provided medical coverage for >99.6% of Taiwan’s residents [22]; because of this high coverage rate, researchers can employ NHIRD data in population-based research with nationwide participant inclusion. NHIRD data are encrypted, and the database contains comprehensive medical claims data of NHI beneficiaries in electronic format, including information regarding disease diagnostic codes (*International Classification of Diseases*, *Ninth Revision*, *Clinical Modification* [ICD-9-CM]) used at the time of ambulatory or inpatient care, examinations and surgeries received, drug prescription details, area of residence, and monthly income level—these data are generated during insurance reimbursement [22].

The data used in this study were obtained from the Longitudinal Health Insurance Database 2005 (LHID2005), a representative subdataset of the NHIRD comprising claims data (for the period 1997–2013) of 1 million insurance beneficiaries randomly selected from the NHIRD’s 2005 Registry of Beneficiaries through systematic sampling; the LHID represents roughly 5% of all Taiwanese residents [23]. No significant differences were found to exist in terms of sex, age, or health care costs between the sample group derived from the LHID2005 and all beneficiaries in the NHIRD [23].

### Study participant selection

**Fig 1:**
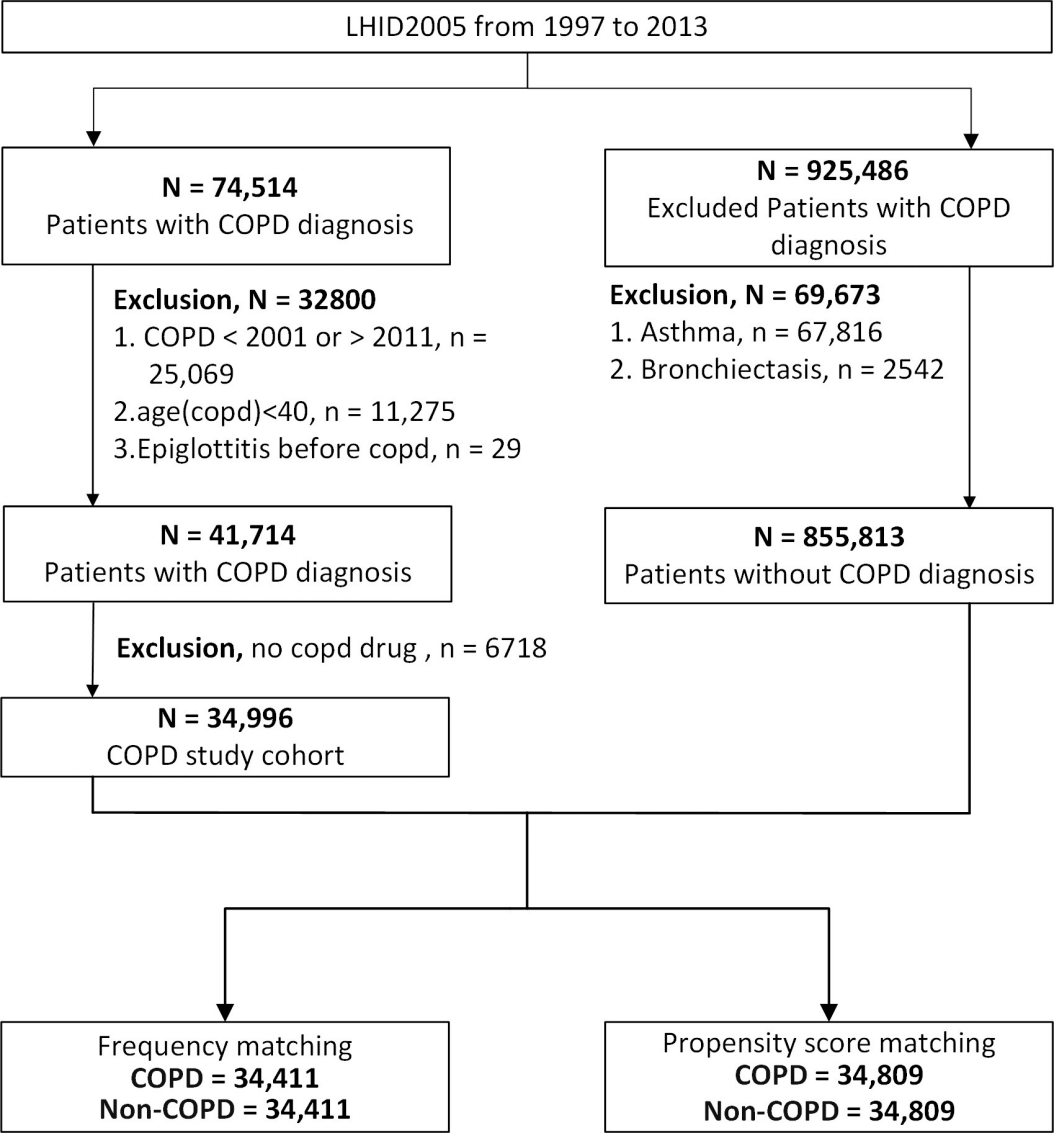
Schematic of the participant enrollment process in this study. Abbreviations: COPD, chronic obstructive pulmonary disease; LHID2005, Longitudinal Health Insurance Database 2005.

Fig 1 illustrates this study’s patient enrollment process. From the LHID2005, we identified patients aged ≥ 40 years newly diagnosed as having COPD (indicated by ICD-9-CM codes 491.xx, 492.xx, and 496) between January 1, 2001, and December 31, 2011; they were enrolled in the study cohort. A patient with COPD was included only if 1) the ICD-9-CM codes for this disease were recorded in more than three ambulatory care claims or in the inpatient setting [24] and 2) medication prescription for COPD was provided two or more times; relevant medications were long- and short-acting beta 2-agonists, long-acting beta 2-agonists combined with inhaled corticosteroids, long- and short-acting muscarinic antagonists, a short-acting muscarinic antagonist combined with short-acting beta 2-agonist, xanthine agents, and phosphodiesterase-4 inhibitors [25–27]. We used these strict inclusion criteria to enhance the accuracy and reliability of the diagnosis of COPD in our enrolled patients. To ensure that for every patient, there was a minimum 2-year follow-up duration, we omitted patients who received a COPD diagnosis after 2011. Those receiving an epiglottitis diagnosis before receiving their COPD diagnosis were also excluded to enhance result validity in terms of the susceptibility of patients with COPD to epiglottitis.

Patients without COPD and other small airway diseases (indicated by ICD-9-CM codes 494.xx [bronchiectasis] and 493.xx [asthma]) were randomly selected from the LHID2005 to establish our comparison cohort. In the matching process, we used both the frequency matching (FM) and propensity-score matching (PSM) to examine our hypothesis. In the FM, we matched each patient with COPD with one randomly selected non-COPD patient from the dataset in regards age, sex, monthly income level, and area of residence. The date of enrollment for included patients with COPD was the COPD diagnosis date; for the matched non-COPD cohort, the index date was the same as that of the patient with COPD they were matched with. In addition, we performed PSM by age, sex, monthly income level, area of residence and medical comorbidities listed in the next paragraph.

### Outcome and covariate measurements

The main outcome in both cohorts was an acute epiglottitis diagnosis (indicated by ICD-9-CM codes 464.3, 464.30, and 464.32) according to the diagnosis coded by the otolaryngologist; diagnoses were confirmed following a minimum of three outpatient claims or one inpatient claim. We followed-up the patients until the occurrence of outcome, the period of investigation ended (December 31, 2013) or death, which was regarded as withdrawal from the NHI program. Therefore, for patients with epiglottitis, only the first outcome occurrence had been recorded.

We retrieved additional comorbidities associated with epiglottitis from inpatient and ambulatory claims data, and we assessed these data in both cohorts [9,10]. The following comorbidities were considered: chronic liver disease (CLD, ICD-9-CM codes 571.xx), coronary artery disease (CAD, ICD-9-CM code 410–414), gastroesophageal reflux disease (GERD, ICD-9-CM codes 530.11, 530.81, 530.85), hypertension (HTN, ICD-9-CM codes 401–405), diabetes mellitus (DM, ICD-9-CM codes 250.xx), alcohol dependence and abuse (ICD-9-CM codes 303, 303.xx, 305.0, and 305.0x), autoimmune diseases (ICD-9-CM codes 714, 710.0, 358.0, 696, 696.1, 696.0, 710.1, 340.0, 710.2, 242, 242.01, 720, 136.1, 245.2, 364.0, 364.01, 710.3, and 710.4), corrosive injury of the upper digestive tract (ICD-9-CM codes 947.0–947.3), and upper gastrointestinal tract cancer (ICD-9-CM codes 141–151). These comorbidities were analyzed as binominal variables. To be included, a particular comorbidity had to have occurred in an inpatient setting or be noted in at least three ambulatory care claims. We identified the comorbidities of each individual prior to the index date or matched index date.

### Statistical analysis

The mean and standard deviation is used to present continuous variable distributions, whereas and frequency and percentage are employed for categorical variables. To compare the demographic characteristics and comorbidities of those with COPD and their comparison cohort in FM, descriptive statistics were employed. The independent Student *t* and Pearson chi-square tests were utilized for analyzing categorical and continuous data, respectively. To evaluate the balance in measured baseline covariates in PSM, we calculated the absolute standardized mean differences (ASMD) for the matched cohort [28]. Previous study proposed that an ASMD of less than 0.1 suggested the balance in baseline covariates [29]. To estimate the cumulative epiglottitis incidence in the two cohorts, the Kaplan–Meier method was employed; intercurve differences were evaluated using the two-tailed log-rank test. The Cox proportional hazard model was employed to calculate the adjusted hazard ratio (aHR) as well as the corresponding 95% confidence interval (CI) for matched-pair epiglottitis diagnosis. Furthermore, subgroup analysis and sensitivity testing were conducted to examine the stability of COPD’s effect on epiglottitis incidence. To evaluate the effects of modifiers, age-, sex-, and comorbidity-stratified subgroup analyses were conducted. We performed statistical analyses with SAS version 9.4 (SAS Inc., Cary, NC, USA); two-sided *p* < 0.05 indicated statistical significance.

## Results

Table 1 lists the sociodemographic characteristics, epiglottitis incidence, and comorbidities of COPD and non-COPD cohorts in the FM and PSM. In the FM, 34,411 patients with COPD and an equal number without COPD were included in this study. After matching was conducted for sex, age, monthly income level, and area of residence, the results indicated that relative to those in the non-COPD cohort, enrollees with COPD were more likely to have epiglottitis (*p* = 0.01, Table 1). In the COPD cohort, the prevalence of CLD, GERD, DM, CAD, HTN, alcohol dependence and abuse, corrosive injury to the upper digestive tract, upper gastrointestinal tract cancer, and autoimmune diseases was significantly higher than the equivalent prevalence in the non-COPD cohort. With the 1:1 PSM by criteria, there were 34,809 patients with COPD and 34,809 patients in the comparison cohort, and the baseline characteristics were balanced between the groups except for the GERD (ASMD = 0.148).

**Table 1:** Baseline characteristics of study participants.

	Frequency matching					Propensity score matching				
	COPD (N = 34,411)		Non-COPD (N = 34,411)			COPD (N = 34,809)		Non-COPD (N = 34,809)		
Variables	n	%	n	%	*p*-value^a^	n	%	n	%	ASMD ^b^
Sex					1.000					0.034
Men	20448	59.4	20448	59.4		20790	59.7	20499	58.9	
Women	13963	40.6	13963	40.6		14019	40.3	14310	41.1	
Age (years)					1.000					0.011
40–64	14671	42.6	14671	42.6		14671	42.2	14438	41.5	
≥65	19740	57.4	19740	57.4		20138	57.9	20371	58.5	
Monthly income (NTD)					1.000					
0	12215	35.5	12215	35.5		12370	35.5	12487	35.9	0.002
1–15840	8403	24.4	8403	24.4		8578	24.6	8559	24.6	<0.001
15841–25000	10557	30.7	10557	30.7		10611	30.5	10655	30.6	0.005
≥25001	3236	9.4	3236	9.4		3250	9.3	3108	8.9	0.021
Area of residence					1.000					
1(City)	7927	23.0	7927	23.0		7984	22.9	8195	23.5	0.029
2	15236	44.3	15236	44.3		15339	44.1	15297	44.0	0.005
3	7232	21.0	7232	21.0		7357	21.1	7315	21.0	0.008
4 (Village)	4016	11.7	4016	11.7		4129	11.9	4002	11.5	0.017
Epiglottitis	60	0.2	35	0.1	0.01	60	0.2	43	0.1	0.002
Comorbidities										
CLD	6641	19.3	4424	12.9	<0.001	6653	19.1	6587	18.9	0.009
HTN	18997	55.2	13943	40.5	<0.001	19241	55.3	19644	56.4	0.023
GERD	1467	4.3	599	1.7	<0.001	1319	3.8	819	2.4	0.148
DM	7645	22.2	5997	17.4	<0.001	7726	22.2	7804	22.4	0.006
CAD	10047	29.2	5981	17.4	<0.001	10149	29.2	9957	28.6	0.024
Corrosive injury of digestive tract	38	0.1	14	0.0	<0.001	38	0.1	26	0.1	0.021
Alcohol dependence	301	0.9	103	0.3	<0.001	297	0.9	234	0.7	0.021
Autoimmune diseases	1557	4.5	1009	2.9	<0.001	1562	4.5	1339	3.9	0.042
Upper GI tract cancer	463	1.4	303	0.9	<0.001	461	1.3	405	1.2	0.006

Abbreviations: ASMD, absolute standardized mean difference; CAD, coronary artery disease; CLD, chronic liver disease; COPD, chronic obstructive pulmonary disease; DM, diabetes mellitus; ESRD, end-stage renal disease; GERD, gastroesophageal reflux disease; GI, gastrointestinal; HTN, hypertension; NTD, New Taiwan dollar.

^a^ Pearson chi-squared tests.

^b^ Absolute standardized mean difference.

Table 2 lists the overall epiglottitis incidence as well as that for the <1, 1–5, and ≥ 5 year follow-up periods. The mean (standard deviation) follow-up periods were 7.3 (3.6) and 7.6 (3.4) years for those with COPD and their non-COPD matches, and the incidence of epiglottitis was 24.0 and 13.4 per 100,000 person-years, respectively. Therefore, the COPD cohort’s incidence of epiglottitis was significantly higher (*p* = 0.006); the mean duration from COPD diagnosis to epiglottitis diagnosis was 4.7 ± 3.2 years. In the FM, the overall epiglottitis incidence rate ratio (IRR) of the COPD group was 1.81 when compared with the non-COPD group (95% CI: 1.19–2.73, *p* = 0.006); at ≥ 5 years of follow-up, the IRR was even higher (IRR = 2.96; 95% CI: 1.44–6.10, *p* = 0.003; Table 2). Similarly, in the PSM, the overall epiglottitis IRR of the COPD group was 1.59 (95% CI: 1.07–2.35, *p* = 0.021) when compared with the non-COPD group, which was even higher at ≥ 5 years of follow-up (IRR = 3.35; 95% CI: 1.63–6.89, *p* = 0.001; Table 2).

**Table 2:** Overall epiglottitis incidence in COPD and non-COPD cohorts.

	COPD				non-COPD					
	N	Epiglottitis	PYs	†Rate	N	Epiglottitis	PYs	†Rate	IRR (95% CI)	*p* value
Frequency matching										
Overall	34411	60	249925.1	24.0	34411	35	262131.8	13.4	1.81 (1.19–2.73)	0.006
Follow years										
< 1	34411	7	33659.8	20.8	34411	9	34208.4	26.3	0.79 (0.29–2.12)	0.614
1–5	33048	25	116024.1	21.5	33986	16	121812.4	13.1	1.64 (0.88–3.07)	0.122
≥ 5	23787	28	100241.1	27.9	25180	10	106111.0	9.4	2.96 (1.44–6.10)	0.003
Propensity score matching										
Overall	34809	60	252178.4	23.8	34809	43	286752.5	15.0	1.59(1.07–2.35)	0.021
Follow years										
< 1	34809	7	34017.2	20.6	34809	9	34505.9	26.1	0.79(0.29–2.12)	0.638
1–5	33382	25	117107.4	21.3	34212	24	131463.3	18.3	1.17(0.67–2.05)	0.584
≥ 5	23999	28	101053.8	27.7	31505	10	120783.3	8.3	3.35(1.63–6.89)	0.001

Abbreviations: COPD, chronic obstructive pulmonary disease; IRR, incidence rate ratio; PYs, person-years.

*Note*.

†Rate: Per 100,000 person-years; IRR was compared using Poisson regression.

**Fig 2:**
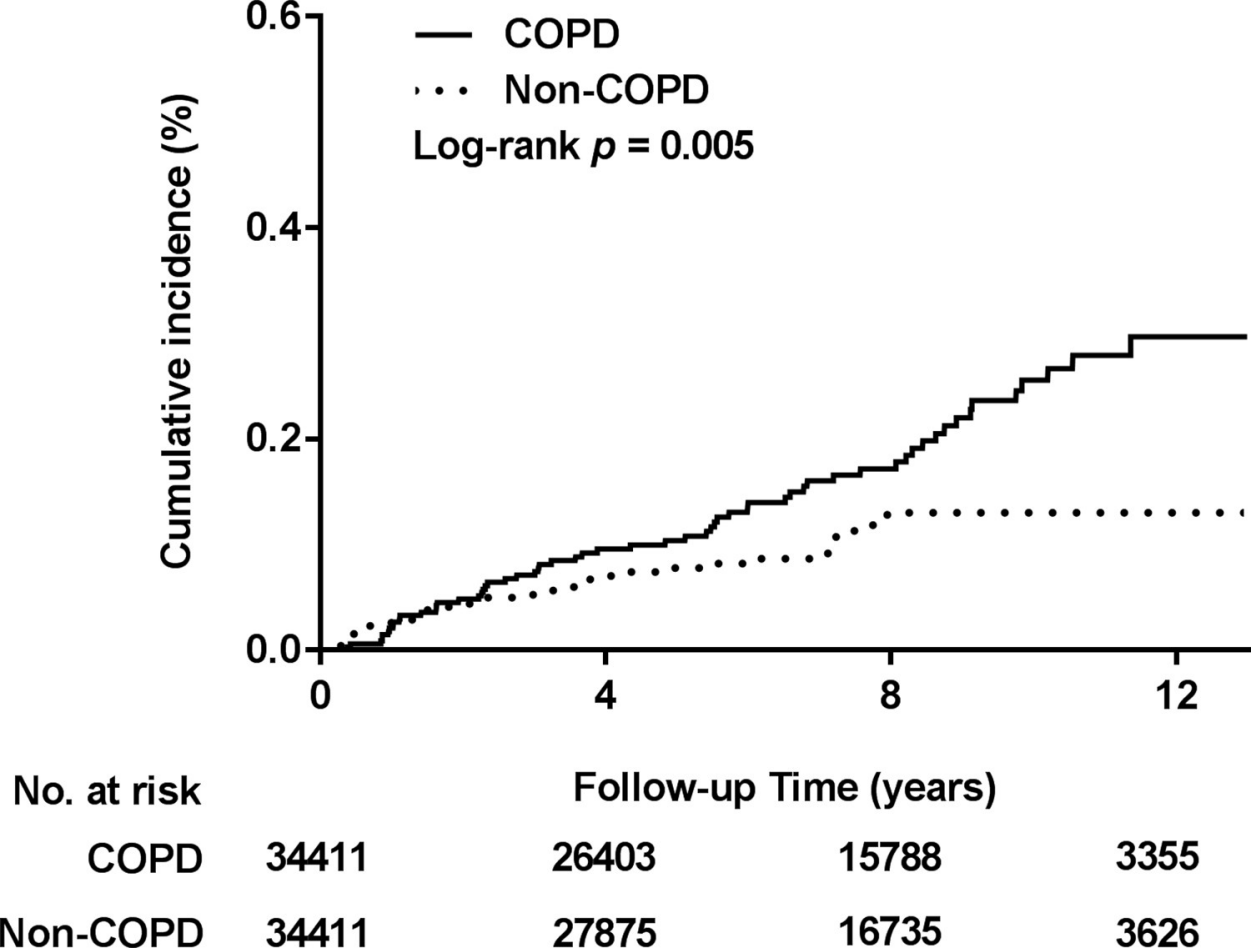
Cumulative incidence of epiglottitis for COPD versus non-COPD. The COPD group had significantly higher cumulative incidence of epiglottitis (log-rank test, *p* = 0.003).

According to the Kaplan–Meier and log-rank test results, the COPD cohort had significantly higher cumulative epiglottitis incidence over the observation period (*p* = 0.005; Fig 2). Table 3 reveals the results of Cox proportional hazard regression analysis, in which the epiglottitis risks of the COPD and matched cohorts were compared. After sex, age, region of residence, and monthly income level were adjusted for, the epiglottitis risk was found to be 1.79-fold (95% CI: 1.18–2.72; *p* = 0.006) greater in the COPD cohort in the FM. Moreover, the full model in which the aforementioned covariates and all comorbidities were adjusted for revealed consistent results (aHR: 1.76; 95% CI: 1.15–2.70; *p* = 0.009). The results of the sensitivity analysis, in which we added every selected comorbidity to the main model, are presented in Table 3 and indicated that the effect of COPD on epiglottitis was consistent and significant. The results of subgroup analyses revealed that the effect of COPD remained significant for the 40- to 64-year age group and for men but not women. In the PSM, both the main model and full model revealed that COPD was correlated with increased risk of epiglottitis, but with marginal statistical significance (aHR: 1.50; 95% CI: 0.99–2.28; *p* = 0.054 and aHR: 1.50; 95% CI: 0.99–2.29; *p* = 0.057, respectively).

**Table 3:** Multivariable Cox proportional hazard model of the association between epiglottitis and potential risk factors.

Variables	Adjusted HR	95% CI	*p* value
**Frequency matching**			
Main model^a^	1.79	(1.18−2.72)	0.006
Full model^b^	1.76	(1.15−2.70)	0.009
**Sensitivity analysis** ^**c**^			
Main model + CLD	1.80	(1.18−2.73)	0.006
Main model + HTN	1.72	(1.13−2.62)	0.012
Main model + GERD	1.76	(1.16−2.67)	0.008
Main model + DM	1.76	(1.16−2.68)	0.008
Main model + CAD	1.83	(1.20−2.78)	0.005
Main model + corrosive injury of upper digestive tract	1.79	(1.18−2.72)	0.006
Main model + alcohol dependence	1.77	(1.16−2.68)	0.008
Main model + autoimmune diseases	1.81	(1.19−2.75)	0.005
Main model + upper GI tract cancer	1.79	(1.18−2.72)	0.006
**Subgroup effects** ^**d**^			
Sex			
Male	1.72	(1.01−2.93)	0.045
Female	1.91	(0.97−3.75)	0.060
Age (years)			
40–64	2.05	(1.12−3.73)	0.019
≥65	1.57	(0.88−2.81)	0.130
CLD			
Yes	1.57	(0.49−5.01)	0.450
No	1.83	(1.17−2.86)	0.008
HTN			
Yes	1.58	(0.85−2.92)	0.147
No	1.89	(1.06−3.35)	0.030
GERD			
Yes	1.04	(0.11−9.17)	0.975
No	1.78	(1.17−2.72)	0.008
DM			
Yes	1.06	(0.45−2.53)	0.893
No	2.03	(1.26−3.27)	0.004
CAD			
Yes	1.47	(0.51−4.19)	0.476
No	1.92	(1.22−3.03)	0.005
Alcohol dependence^e^			
Yes	-	-	-
No	1.74	(1.14−2.65)	0.010
Autoimmune diseases^f^			
Yes	-	-	-
No	1.79	(1.18–2.72)	0.006
**Propensity score matching**			
Main model^a^	1.50	(0.99−2.28)	0.054
Full model^b^	1.50	(0.99−2.29)	0.057

Abbreviations: CAD, coronary artery disease; CI, confidence interval; CLD, chronic liver disease; DM, diabetes mellitus; GERD, gastroesophageal reflux disease; GI, gastrointestinal; HR, hazard ratio; HTN, hypertension.

^a^ Main model was adjusted for sex, age, urbanization level, and income.

^b^ Full model was adjusted for sex, age, urbanization level, income, and all comorbidities.

^c^ The models were adjusted for covariates in the main model as well as each comorbidity.

^d^ Models for subgroup analysis were adjusted for sex, age, urbanization level, income, and comorbidities.

^e,f^ For subgroup analysis under main model for patients with alcohol dependence and autoimmune diseases, the case numbers are insufficient for statistical analysis.

## Discussion

Given that patients with epiglottitis may develop acute and life-threatening airway obstruction, underlying diseases that may exacerbate the clinical course of epiglottitis must be recognized early and properly treated [30]. Both Western and Eastern studies have reported that 13%–15.4% of patients with epiglottis have comorbid pulmonary disease [9,20], and according to our review of the literature, the present study is the first to demonstrate an association between COPD and elevated risk of epiglottitis in adults. In clinical studies, researchers face difficulties in recruiting a sufficient number of participants with adequate follow-up time to be able to determine the long-term incidence of epiglottitis following a COPD diagnosis. Because a nationwide database was employed in the present study, a sufficient number of COPD and epiglottitis cases could be identified with negligible selection bias because the vast majority of health care services received by Taiwanese residents are covered by its NHI program [22]. In addition, the long observation periods were sufficient for detecting trends in epiglottitis risk in the two cohorts. To clarify the event sequence, a patient was included only if they had received a COPD diagnosis before an epiglottitis diagnosis. The total epiglottitis incidence rate in the COPD cohort was higher than in the comparison cohort, with an IRR of 1.81 (24.0 vs. 13.4/100 000 person-years, *p* = 0.006) in the FM and an IRR of 1.59 (23.8 vs. 15.0/100 000 person-years, *p* = 0.021) in the PSM. Moreover, the overall epiglottitis IRR of the COPD group was even higher at ≥ 5 years of follow-up in both FM and PSM (IRR = 2.96 and 3.35, respectively). According to the log-rank test findings, the cumulative epiglottitis incidence in the COPD cohort was significantly higher (*p* = 0005). To evaluate the effects of potential confounders, the multivariate Cox proportional hazard model was employed to compare the two cohorts in terms of outcome. After adjustment for sex, age, region of residence, monthly income level, and comorbidities (CLD, DM, HTN, GERD, CAD, corrosive injury of upper digestive tract, autoimmune diseases, upper gastrointestinal tract cancer, and alcohol dependence), those with COPD had a 1.76 times higher risk of epiglottitis than the non-COPD cohort in the FM and 1.50 times higher risk of epiglottitis than the comparison cohort in the PSM. Of note, although the trend of COPD and increased epiglottitis risk was observed in both the FM and PSM, the results of multivariable Cox proportional hazard analysis did not reach statistical significance in the PSM. Our findings, therefore, should be interpreted cautiously because of the observed wide CI, which may be resulted from the enrollment of small numbers of patients with epiglottitis. Moreover, we conducted both sensitivity tests and subgroup analyses to verify the effect of COPD on epiglottitis in the FM. The results of sensitivity tests remained significant and constant after adding different comorbidities to the model and the effect was validated in men and those in the 40- to 64-year subgroups. In the present, we used the FM and PSM to examine our hypothesis and demonstrate the novel link between epiglottitis and COPD; these findings may extend knowledge on the predisposing factors and pathophysiology of epiglottitis.

In the subgroup analysis, an association was discovered between COPD and elevated risk of epiglottitis in men and those aged 40–64 years. A study reported a male predilection of 2.5:1 for adult epiglottitis [31]. COPD also exhibits sex differences with respect to symptoms, comorbidities, and phenotypes [32], and these sex differences may influence the association of epiglottitis with COPD. Several factors may also contribute to this observation, including personal habits, socioeconomic status, and hormone differences [33,34]. In Taiwan, far fewer women smoke than men (odds ratio, 17.9; 95% CI, 15.4–20.8) [35], and the cigarette smoking has been recognized as the most important causative factor of COPD [36]. Therefore, the increased risk of COPD in men may contribute to our finding. The sex differences may also be partially because of the role of androgens take in the regulation of respiratory disease resistance genes and immune response [37]. The diagnosis of adult epiglottitis is most likely at 42 to 48 years of age [31], and we observed a significantly higher epiglottitis risk in the 40–64-year age subgroup but not in those aged ≥65 years. Patients who are 40–64 years old are generally healthy and have fewer comorbidities than those who are older; therefore, the impact of COPD may be accentuated in this population. Instead, increased vulnerability to comorbidities and infections due to age-related changes may decrease the impact of COPD on epiglottitis in individuals >65 years old. Overall, physicians should be mindful of the occurrence of epiglottitis in patients with COPD, particularly in men and those aged 40–64 years.

The results of this study suggested that COPD is a potential risk factor for epiglottitis, and the development of epiglottitis in patients with COPD may have prognostic and therapeutic implications. The upper respiratory tract infection is a common causative agent of acute exacerbation of COPD [38] which is a significant contributor to the mortality and morbidity associated with COPD [39]. Given that the epiglottitis is an acute and potentially life-threatening upper respiratory infection, the timely treatment of epiglottitis in patients with COPD may prevent not only the need for airway intervention but also the severe exacerbations of COPD [40]. On the other hand, the underlying COPD may aggravate the clinical course of epiglottitis. Shah et al. conducted a retrospective review of a nationwide inpatient dataset from the United States and found that 13% of patients with epiglottitis had underlying pulmonary disease that had clearly exacerbated their clinical course [9]. In patients with COPD, changes in upper-airway-protective mechanisms, laryngeal narrowing during expiration, and ventilatory adaptations may all contribute to the worsening of epiglottitis [41,42]. A study also reported the effects of COPD on patients who underwent laryngectomy for laryngeal cancer, including greater hospitalization length and cost and more postoperative pulmonary complications [43]. The aforementioned findings suggest that adequate treatment of underlying COPD may lead to improved outcomes in patients with COPD–epiglottitis. Nevertheless, the mechanism of the association between COPD and epiglottitis remains unclear because of the rarity of epiglottitis. Factors possibly explaining the significant COPD–epiglottitis association observed in the current study are impaired regulatory T cell generation and functions [44], leptin deficiency [45], and smoking-induced structural and functional immune alteration [46]; these factors all increase COPD patients’ susceptibility to epiglottitis. Autoimmunity may also mediate the association of epiglottitis with COPD as a shared pathophysiology. Autoimmunity has been proposed as one of the pathophysiologies of COPD [47], and noninfectious epiglottitis caused by autoimmunity with laryngeal involvement has been reported [48]. Furthermore, *S*. *pneumoniae*, *Moraxella catarrhalis*, and *Haemophilus influenzae* are the principal pathogens leading to acute worsening of COPD episodes [49]; the aforementioned pathogens are also the most frequent bacterial pathogens of epiglottitis in both vaccinated children [6] and adults [50]. The studies highlighted in this section provide evidence to support the association of COPD with epiglottitis, and the underlying pathophysiological mechanisms warrant further investigation.

This study is the first to recognize COPD’s influence on the risk of epiglottitis development. The representative population, large sample, and long observation period enabled us to investigate a disease with low incidence with high statistical power and risk appraisal precision. However, this study has several limitations. First, although we used strict criteria to enhance the diagnostic accuracy for COPD and epiglottitis, the database we employed did not include the clinical findings of epiglottitis, history of allergy as well as results of laboratory examinations (such as IgE level), image studies, and pulmonary function tests. The heterogeneous severity of epiglottitis and COPD may have led to underestimation of the correlation between these two diseases. Second, we could not identify the definite etiology of epiglottitis through use of the ICD-9-CM codes recorded in the database. Also, the causative pathogens of epiglottitis cannot be identified in this study because the culture reports are not available in the Taiwan NHIRD. In addition, the misclassifications of epiglottitis with anaphylaxis and asthma with COPD are possible in this study setting. Therefore, we could not identify a correlation between COPD and epiglottitis caused by different etiologies. However, the pathophysiological elements involved in the underlying inflammation found in epiglottis [21] were also found in the dysregulated inflammation of the COPD [17], which may explain the results obtained in this study. Third, the retrospective study design had inherent limitations, such as missing or unavailable data. In addition, degrees of bias may develop because several suspected risk factors of epiglottitis, such as the personal habits of cigarette smoking and high body mass index, were unavailable in the database we used, which led to confounding factors that are difficult to be adjusted for. However, after reviewing 6072 patients with epiglottitis, Suzuki et al. found no significant association between smoking habit and epiglottitis [20]. Therefore, the association between COPD and epiglottitis may be less likely to be influenced by the presence of smoking habits. To further validate our study results, future prospective studies should investigate the associations of epiglottitis with subcategories of COPD or exacerbating disease as well as the potential impact of COPD medication for the development of epiglottitis.

## Conclusion

This cohort study using nationwide population-based data is the first to indicate that COPD may be a risk factor for epiglottitis in adults, particularly in those aged 40–64 years and men. To prevent life-threatening complications, make a timely diagnosis, and provide an adequate intervention, physicians should remain cognizant of the fact that the risk of epiglottitis in patients with COPD is likely increased. Further large-scale, prospective studies should be conducted to verify our findings.
